# Development of a Mortality Prediction Tool in Pediatric Severe Traumatic Brain Injury

**DOI:** 10.1089/neur.2020.0039

**Published:** 2021-02-23

**Authors:** Kawmadi Abeytunge, Michael R. Miller, Saoirse Cameron, Tanya Charyk Stewart, Ibrahim Alharfi, Douglas D. Fraser, Janice A. Tijssen

**Affiliations:** ^1^ Schulich School of Medicine and Dentistry, Western University, London, Ontario, Canada.; ^2^ Department of Paediatrics, Western University, London, Ontario, Canada.; ^3^ Children's Health Research Institute, London, Ontario, Canada.; ^4^ Lawson Health Research Institute, London, Ontario, Canada.; ^5^ Department of Pediatric Critical Care, Children's Hospital, King Fahad Medical City, Riyadh, Saudi Arabia.; ^6^ Department of Clinical Neurological Sciences, Western University, London, Ontario, Canada.

**Keywords:** critical care, mortality, pediatric, prognosis, traumatic brain injury

## Abstract

Severe traumatic brain injury (sTBI) is a leading cause of pediatric death, yet outcomes remain difficult to predict. The goal of this study was to develop a predictive mortality tool in pediatric sTBI. We retrospectively analyzed 196 patients with sTBI (pre-sedation Glasgow Coma Scale [GCS] score <8 and head Maximum Abbreviated Injury Scale (MAIS) score >4) admitted to a pediatric intensive care unit (PICU). Overall, 56 patients with sTBI (29%) died during PICU stay. Of the survivors, 88 (63%) were discharged home, and 52 (37%) went to an acute care or rehabilitation facility. Receiver operating characteristic (ROC) curve analyses of admission variables showed that pre-sedation GCS score, Rotterdam computed tomography (CT) score, and partial thromboplastin time (PTT) were fair predictors of PICU mortality (area under the curve [AUC] = 0.79, 0.76, and 0.75, respectively; *p* < 0.001). Cutoff values best associated with PICU mortality were pre-sedation GCS score <5 (sensitivity = 0.91, specificity = 0.54), Rotterdam CT score >3 (sensitivity = 0.84, specificity = 0.53), and PTT >34.5 sec (sensitivity = 0.69 specificity = 0.67). Combining pre-sedation GCS score, Rotterdam CT score, and PTT in ROC curve analysis yielded an excellent predictor of PICU mortality (AUC = 0.91). In summary, pre-sedation GCS score (<5), Rotterdam CT score (>3), and PTT (>34.5 sec) obtained on hospital admission were fair predictors of PICU mortality, ranked highest to lowest. Combining these three admission variables resulted in an excellent pediatric sTBI mortality prediction tool for further prospective validation.

## Introduction

Severe traumatic brain injury (sTBI) is defined by a Glasgow Coma Scale (GCS) score ≤8, and is a leading cause of pediatric death in developed countries.^1,2^ Despite the prevalence of sTBI, related outcomes remain difficult to predict. Given that sTBI is highly associated with mortality,^1–3^ having a reliable method to predict mortality shortly after injury would be invaluable for health care providers and caregivers.

A number of factors have been associated with mortality and poor functional outcome in pediatric patients with sTBI, including low GCS score,^4–12^ younger age,^6^ absent pupillary response,^7,8,10–13^ a higher Injury Severity Score (ISS),^5^ and the presence of hypernatremia,^8^ hypoxia,^9,13^ or hypotension.^5,7,10,11^ Altered laboratory values that have also been studied as prognostic factors include increased international normalized ratio (INR),^14–16^ partial thromboplastin time (PTT),^12,16^ thrombocytopenia,^16^ and hyperglycemia in pediatric sTBI,^6,17^ and increased neutrophil-to-lymphocyte ratio (NLR) in adult TBI.^18^ More recently, the Rotterdam computed tomography (CT) score was developed and validated in pediatric sTBI, and includes midline shift, subarachnoid hemorrhage, abnormal basal cisterns, epidural mass lesions, and intraventricular blood.^19–21^

Although several early indicators have been shown to be independently associated with sTBI mortality, appropriate cutoffs and the relative strength of these indicators are unknown. The aim of this study was to develop a mortality prediction tool for pediatric sTBI for prospective validation using hospital admission variables.

## Methods

### Study design and participants

A retrospective cohort study was conducted for patients admitted to the pediatric intensive care unit (PICU) at the Children's Hospital, London Health Sciences Centre (LHSC) with sTBI between January 1, 2000 and December 31, 2015. Research ethics approval was obtained from the Research Ethics Board at Western University. Over the study period, patients with sTBI in our institution were managed as per published guidelines, including placement on a temperature-modulating blanket in supine position, with the head elevated to 30 degrees. Normothermia was targeted in patients not enrolled in the hypothermia trial,^22^ with administration of antipyretics as needed, passive cooling, and/or use of the temperature modulating blanket.

Antibiotics were not routinely administered, except for the inconsistent prophylactic use of cefazolin after intracranial pressure (ICP) monitor placement.^23^ Adequate analgesia and sedation were obtained with opioid and benzodiazepine infusions, respectively. Raised ICP was generally managed as per published guidelines.^24^ If ICP was >20–25 mm Hg for >5 min, or for a rapidly raising ICP: 1) cerebrospinal fluid was drained for 5 min if there was an external ventricular drain *in situ*^23^; 2) mannitol 0.5 g/kg was intravenously (IV) given over 20 min every 6 h as needed; 3) 3% sodium chloride (NaCl) was given in boluses of 1–2 mL/kg over 5 min every 12 h as needed (first dose 2–4 mL/kg); 4) hyperventilation was set to a PaCO_2_ of 35 mm Hg; 5) barbiturate infusion was titrated to ICP and cerebral perfusion pressure (CPP); and 6) neurosurgical consult was sought for potential decompressive craniectomy. Mannitol and 3% NaCl were held if the measured osmolality values were >320 and 360 mOsm/L, respectively. Low CPP secondary to arterial hypotension and without raised ICP was managed with 0.9% NaCl (or colloid) 10 mL/kg IV over 5–30 min as needed, followed by administration of inotropes/vasopressors.

Patients were included in the study if they were <18 years old at the time of injury and sustained an sTBI, defined as a pre-sedation GCS score ≤8 and a head Maximum Abbreviated Injury Scale (MAIS) score ≥4. Patients who died prior to PICU admission were excluded. Demographic and injury data were abstracted from our previously published sTBI database,^7,8,10–12,25,26^ the hospital electronic database, and the paper copy of the patient's hospital chart (Table 1). The collected hospital admission data included age, sex, pre-sedation GCS score, ISS, and MAIS score. Pre-sedation GCS score was determined at the scene, referring hospital, or on arrival to the LHSC trauma center. The Rotterdam CT score was calculated based on brain imaging findings on hospital arrival^19^; these included appearance of basal cisterns, degree of midline shift, presence of epidural mass lesions, intraventricular hemorrhage, and subarachnoid hemorrhage. All blood hematological values were obtained in the trauma resuscitation room at the time of patient arrival, except for the blood glucose level (mmol/L) and the NLR (measured as neutrophil count divided by lymphocyte count), which were the peak values within the first 24 h of hospital admission.

**Table 1.** Bivariate Analyses of Demographic Characteristics, Laboratory Data, and CT Findings of Patients Who Died and Patients Who Survived in the PICU

Variable	PICU mortality (*n* = 56)	PICU survival (*n* = 140)	*P*-value
Age, years	14 (1, 17)	12 (6, 16)	0.792
Male	37 (66.1)	94 (67.1)	0.886
Pre-sedation GCS score	3 (3, 4)	6 (4, 7)	**<0.001**
ISS	33 (27, 43)	30 (26, 38)	**0.005**
Injury profile			
MAIS head	5 (5, 5)	5 (5, 5)	**0.026**
MAIS face	1 (1, 2)	1 (1, 2)	0.308
MAIS neck	1 (1, 1)	3 (1, 3)	0.248
MAIS thorax	3 (2, 3)	3 (2, 3)	**0.029**
MAIS abdomen	2 (2, 2)	2 (2, 3)	0.204
MAIS spine	2 (2, 3)	2 (1.5, 2)	**0.020**
MAIS extremities	2 (2, 3)	2 (2, 3)	0.349
MAIS external	1 (1, 1)	1 (1, 1)	0.573
Mechanism of injury			0.153
MVC	39 (69.6)	99 (70.7)	0.882
Fall	2 (3.6)	15 (10.7)	0.160
Intentional	10 (17.9)	12 (8.6)	0.063
Other	5 (8.9)	14 (10.0)	0.819
Rotterdam CT score	3 (3, 5)	2 (2, 3)	**<0.001**
CT characteristics			
SAH	29 (54.7)	53 (39.0)	**0.050**
IVH	17 (32.1)	26 (19.1)	0.056
Absent basal cisterns	24 (45.3)	8 (5.8)	**<0.001**
Compressed basal cisterns	7 (13.2)	6 (4.3)	**0.049**
Epidural mass lesion	50 (90.9)	121 (86.4)	0.391
Midline shift >5 mm	9 (17.0)	13 (9.4)	0.143
Laboratory values			
Platelets, ^*^10^9^/L	138.0 (85.0, 249.0)	198.5 (151.0, 254.8)	**0.001**
NLR^a^	6.5 (2.2, 13.5)	10.4 (5.2, 17.0)	**0.010**
INR, [PTt/PTn]^ISI^	1.5 (1.0, 2.0)	1.0 (1.0, 1.0)	**<0.001**
PTT, sec	42 (32, 68)	31 (28, 36)	**<0.001**
Glucose, mmol/L^a^	10.4 (7.7, 14.1)	7.8 (6.8, 9.6)	**<0.001**

^a^ Highest value in first 24 h (all other values are taken from admission blood draw).

Continuous variables are presented as median (interquartile range) and categorical variables as *n* (%).

Bold values indicate statistical significance of *p* ≤ 0.05.

CT, computed tomography; GCS, Glasgow Coma Scale; INR, international normalized ratio; ISS, Injury Severity Score; IVH, intraventricular hemorrhage; MAIS, Maximum Abbreviated Injury Scale; MVC, motor vehicle collision; NLR, neutrophil to lymphocyte ratio; PICU, pediatric intensive care unit; PTT, partial thromboplastin time; SAH, subarachnoid hemorrhage.

The primary outcome of this study was PICU mortality. A secondary outcome was discharge disposition of survivors (home, acute care facility, or chronic rehabilitation center).

### Statistical analysis

Demographics, trauma severity scores, and brain imaging findings were summarized using descriptive statistics. Due to deviations from normalcy, continuous variables were presented as median and interquartile ranges. Categorical variables were presented as frequencies and percentages. Comparisons between patients who survived and died were examined with two-tailed Mann-Whitney U tests and Pearson chi-square tests (or Fisher's exact tests when appropriate) for continuous and categorical variables, respectively. A multiple regression was conducted to assess whether temporal trends in mortality were statistically significant.

Receiver operating characteristic (ROC) curves were estimated to determine sensitivity and specificity of all continuous variables for predicting mortality. Discharge disposition was dichotomized into discharge home or acute care facility/rehabilitation center, with the latter being considered unfavorable; ROC curves were completed for continuous variables with discharge disposition as a secondary outcome. The area under the curve (AUC) was calculated for each variable, with an AUC >0.7 considered as acceptable.^27^ The coordinates of the curves were then analyzed using Youden's index to identify the cutoff values based on the highest sensitivity and specificity for predicting mortality and discharge disposition. A logistic regression analysis was also conducted with PICU mortality as the outcome, and variables with an AUC >0.7 entered as predictors; the predicted values from the logistic regression were then saved for use in a ROC curve analysis. All analyses were conducted using SPSS version 25 (IBM Corp., Armonk, NY, USA).

## Results

### Patient characteristics

A total of 196 pediatric patients with sTBI met inclusion criteria. Patient and injury demographics are reported in Table 1. The majority of the patients were male (67%) with a median age of 13 years, and the most common mechanism of sTBI was motor vehicle collision (70%). Fifty-six (29%) patients died in the PICU, and there was no significant temporal trend in rates of death over the study period (*p* = 0.296). Of the patients who survived their PICU admission, 63% were discharged home, 9% were discharged to an acute care facility, and 29% to a chronic rehabilitation facility. For the variables of interest, only 2.1% of data were missing.

Patients who died had a lower pre-sedation GCS score (*p* < 0.001), higher ISS (*p* = 0.005), and higher Rotterdam CT score (*p* < 0.001) (Table 1). Although MAIS head, thorax, and spine were significantly associated with PICU mortality, the median values were the same between groups (medians = 5, 3, and 2, respectively). Absent or compressed basal cisterns were significantly associated with PICU mortality (*p* < 0.001 and *p* = 0.049, respectively). Patients who died had a significantly lower platelet count (*p* = 0.001), lower NLR (*p* = 0.010), higher INR (*p* < 0.001), higher PTT (*p* < 0.001), and higher glucose (*p* < 0.001) (Table 1).

### Mortality prediction

**FIG. 1.**
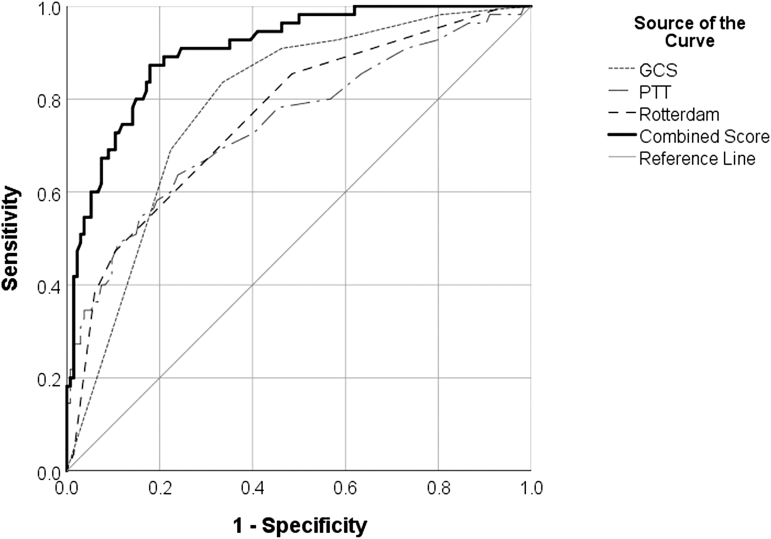
Receiver operating characteristic curves for pre-sedation GCS score (AUC = 0.79), Rotterdam CT score (AUC = 0.76), PTT (AUC = 0.75), and combination of pre-sedation GCS score, Rotterdam CT score, and PTT (0.91) as predictors of PICU mortality. AUC, area under the curve; CT, computed tomography; GCS, Glasgow Coma Scale; PICU, pediatric intensive care unit; PTT, partial thromboplastin time.

Pre-sedation GCS score, Rotterdam CT score, and PTT had AUCs >0.7 (0.79, 0.76, and 0.75, respectively; *p* < 0.001), making these variables fair predictors of mortality (Table 2). INR, glucose, platelets, NLR, ISS, and age were poor predictors of mortality (AUCs = 0.69, 0.69, 0.65, 0.62, 0.61, and 0.51, respectively). Figure 1 depicts ROC curves for pre-sedation GCS score, Rotterdam CT score, and PTT with PICU mortality as the outcome. Sensitivity and specificity were best with a pre-sedation GCS score <4.5 (sensitivity = 0.84, specificity = 0.67), Rotterdam CT score >2.5 (sensitivity = 0.84, specificity = 0.53), and PTT >34.5 sec (sensitivity = 0.69, specificity = 0.67) (Table 2). However, because pre-sedation GCS score and Rotterdam CT scores can only take on integer values, the optimal sensitivity and specificity of the nearest whole number for these variables was found to be best with a pre-sedation GCS <5 (sensitivity = 0.91, specificity = 0.54) and Rotterdam CT score >3 (sensitivity = 0.84, specificity = 0.53). Additionally, a ROC curve was produced with the combined predictive values of pre-sedation GCS score, Rotterdam CT score, and PTT (Fig. 1). The AUC for this combined curve was 0.91, making it an excellent predictor of PICU mortality. This combined tool had a positive predictive value of 94%, and negative predictive value of 67% in predicating PICU mortality.

**Table 2.** Receiver Operating Characteristic Curve Analyses for All Continuous Variables with PICU Mortality as an Outcome

Variable	Area under curve	Standard error	Asymptotic significance	Cutoff	Sensitivity	Specificity
Pre-sedation GCS score	**0.79**	0.04	<0.001	<4.50	0.84	0.67
Rotterdam CT score	**0.76**	0.04	<0.001	>2.50	0.84	0.53
PTT, sec	**0.75**	0.04	<0.001	>34.50	0.69	0.67
INR, [PTt/PTn]^ISI^	0.69	0.05	<0.001	-	-	-
Glucose, mmol/L^a^	0.69	0.05	<0.001	-	-	-
Platelets, ^*^10^9^/L	0.65	0.05	0.002	-	-	-
NLR^a^	0.62	0.05	0.012	-	-	-
ISS	0.61	0.05	0.022	-	-	-
Age, years	0.51	0.05	0.895	-	-	-

^a^ Highest value in first 24 h (all other values are taken from admission blood draw).

Variables are listed from highest area under curve to lowest.

Bold values indicate area under curve >0.7.

CT, computed tomography; GCS, Glasgow Coma Scale; INR, international normalized ratio; ISS, Injury Severity Score; NLR, neutrophil to lymphocyte ratio; PICU, Pediatric Intensive Care Unit; PTT, partial thromboplastin time.

Pre-sedation GCS score, Rotterdam CT score, and PTT score remained significant in predicting PICU mortality when entered as predictors in a logistic regression model (model *p* < 0.001; Hosmer-Lemeshow *p* = 0.641) (Table 3). Specifically, a lower pre-sedation GCS was found to be significantly associated with PICU mortality (odds ratio [OR] = 0.48; 95% confidence interval [CI] = 0.34-0.67, *p* < 0.001), whereas a higher Rotterdam CT score (OR = 2.25; 95%CI = 1.53-3.30, *p* < 0.001) and higher PTT (OR = 1.05; 95% CI = 1.02-1.07, *p* < 0.001) were significantly associated with PICU mortality.

**Table 3.** Logistic Regression of Admission Variables with PICU Mortality as Outcome

Variable	ß	SE	OR	95% CI	*P*-value
Pre-sedation GCS score	-0.74	0.17	0.48	0.34-0.67	**<0.001**
Rotterdam CT score	0.81	0.20	2.25	1.53-3.30	**<0.001**
PTT, sec	0.05	0.01	1.05	1.02-1.07	**<0.001**

Bold values indicate statistical significance of *p* < 0.05.

CI, confidence interval; CT, computed tomography; GCS, Glasgow Coma Scale; PICU, pediatric intensive care unit; OR, odds ratio; PTT, partial thromboplastin time; SE, standard error.

### Discharge disposition prediction

Of the continuous variables examined in ROC curve analyses with discharge to a care facility as the outcome, only Rotterdam CT score had an AUC >0.7 (AUC = 0.71, *p* < 0.001), making it a fair predictor of unfavorable discharge disposition. Pre-sedation GCS score, age, ISS, platelets, INR, PTT, glucose, and NLR were poor predictors of discharge disposition (AUCs = 0.69, 0.66, 0.65, 0.63, 0.62, 0.53, 0.50, and 0.47, respectively). Sensitivity and specificity were best at a Rotterdam CT score >2.5 (sensitivity = 0.71, specificity = 0.67). As this score can only take on an integer value, the sensitivity and specificity of the nearest whole number for this variable was found to be best with a Rotterdam CT score >3.

## Discussion

We report the development of a mortality prediction tool following pediatric sTBI in a cohort of 196 patients. Of these patients, 67% were male and 70% of the injuries resulted from motor vehicle collisions, which is consistent with other epidemiological studies analyzing the demographics and etiologies of sTBI.^2,28,29^ In our cohort, 29% of patients died during their PICU stay, and there was no significant change in rates of death over the study period. This mortality rate likely reflects our strict inclusion criteria (pre-sedation GCS score <8 and head MAIS >4). Given that higher head MAIS has been significantly associated with poorer outcomes in pediatric sTBI, our patient population may represent more severe TBI.^30,31^ Moreover, this mortality rate is comparable to another study utilizing a head MAIS criteria >3, within a population of intubated pediatric sTBI patients with similar ISS to ours.^32^

Our analysis demonstrated that pre-sedation GCS score, Rotterdam CT score, and PTT were fair predictors of mortality following pediatric sTBI, whereas INR, glucose, platelets, NLR, ISS, and age were poor predictors. Predictive values for these former variables were best with cutoffs of pre-sedation GCS score <5, Rotterdam CT score >3, and PTT >34.5 sec. Importantly, all three variables utilized are hospital admission variables, with the pre-sedation GCS score reported on trauma room arrival, the blood drawn in the resuscitation room, and the CT scan obtained prior to PICU admission.

The Rotterdam CT score was also found to be a fair predictor of unfavorable discharge disposition, with a cut-off of Rotterdam CT score >3 best predicting discharge to an acute care facility or chronic rehabilitation center. As patients requiring ongoing care were less functionally independent on discharge, the Rotterdam CT score can serve as a fair surrogate predictor of unfavorable functional outcome on discharge, but more studies are required to confirm this hypothesis.

Our findings on the predictive value of pre-sedation GCS score are consistent with the literature, reporting GCS score to be a powerful and significant independent indicator of mortality and morbidity following sTBI in pediatric populations.^4–6^ Our pre-sedation GCS score cutoff of <5 is congruent with other studies that also report a GCS score threshold of <5 to be a predictor of sTBI mortality in children.^4–6^ By specifying pre-sedation GCS, our study avoids the possibility of including artificially low GCS values for patients whose scores were taken following sedation medication administration, thus making our assessment of injury more accurate.

CT findings are associated with mortality and morbidity outcomes.^10,11,13,19–21^ The Rotterdam CT score has been recently identified as a validated and accurate tool for risk stratification of children with sTBI.^19–21^ The components of this score are based on imaging findings that have been previously established as predictors of poor outcomes in pediatric sTBI of all ages, including subarachnoid hemorrhage and midline shift.^9,11,13,19^ In our study, the Rotterdam CT score was a fair predictor of mortality and unfavorable discharge disposition with a cutoff score >3. This finding is in agreement with a recent study that also reports a Rotterdam CT score of 3 as an acceptable cutoff for predicting mortality in pediatric TBI patients.^20^

Although the exact pathophysiology of trauma-induced coagulopathy remains unclear, abnormalities in laboratory markers of coagulation including INR, PTT, and platelets have been associated with mortality.^14–16^ Injury causes endothelial damage, increased sympathetic drive, and systemic inflammation that may contribute to the observed changes in hemostasis.^16^ Whereas INR and platelets failed to provide a reliable AUC in our ROC curve analysis, we found that a PTT cutoff value >34.5 was a fair predictor of sTBI mortality. This cutoff value is similar to those found in the literature defining acute traumatic coagulopathy in children, ranging from 33 sec to 42.1 sec.^16^ While this cutoff value is above the normal reference range of 20–29 sec for children over 6 months of age, it is within normal range for children under 6 months.^33^ Although not significant in predicting mortality in this study, increasing age has previously been associated with TBI coagulopathy, suggesting that hemostatic dysfunction occurring early after TBI may be better tolerated in early childhood.^16^

Combining pre-sedation GCS score <5, Rotterdam CT score >3, and PTT >34.5 sec resulted in an excellent predictor of mortality (AUC = 0.91) with a positive predictive value of 94%. Compared with published prognostic models in adult sTBI, the AUC of our mortality tool surpasses those reported in the application of the well-recognized International Mission on Prognosis and Analysis of Clinical Trials in TBI (IMPACT),^34,35^ and is comparable to AUCs of machine learning models.^36^ Applying our mortality tool on PICU admission is especially appealing given the lack of prognostic tools in pediatric sTBI. A recent study was published on the use of Base Deficit, International Normalized Ratio, and Glasgow Coma Scale (BIG) score in predicting functional outcome in pediatric patients with TBI presenting to the emergency department; however, it utilized a TBI patient population with milder TBI by including those with a GCS score >8.^37^ Our tool predicted mortality for patients with severe TBI specifically, and with a greater AUC compared with the BIG study.^37^ An AUC of 0.91 is promising, and future efforts should be pursued to validate and translate this tool into the clinical setting.

There were several limitations to our study. First, this was a single-center study; nevertheless, our data should be translatable to other Level 1 trauma centers in developed countries as our study had a sizeable patient population, appropriate statistical power, and used strict inclusion criteria for sTBI and standard definitions for CT findings. Second, our database only captured mortality outcomes occurring during the PICU stay and disposition at hospital discharge. Future research should also focus on determining prognostic reliability of this prediction tool, with or without additional variables, for functional outcomes in survivors (e.g., Glasgow Outcome Score) at specific time-points (e.g., 12 months post-discharge). Finally, the circumstances around each PICU death were not documented, as were the criteria used in withdrawal-of-care cases. Thus, it is possible that the strength of the association between our mortality tool and death was exaggerated, in part, by the unrecognized influence of these variables on subsequent withdrawal of care decision making. Despite these caveats, our mortality prediction tool is feasible early after admission to the hospital, straightforward and easy to apply clinically, and can be prospectively validated prior to widespread utilization.

In summary, a combination of pre-sedation GCS score, Rotterdam CT score, and PTT resulted in an excellent mortality prediction tool following sTBI in a pediatric population. In contrast, INR, glucose, platelets, NLR, ISS, and age were poor predictors of PICU mortality. A pre-sedation GCS score <5, a Rotterdam CT score >3, and PTT >34.5 sec had the greatest sensitivity and specificity for predicting mortality. Future directions should include prospective validation as well as incorporate long-term function at specific time-points as an outcome.

## Abbreviations Used

AUC: area under the curve
BIG: Base Deficit, International Normalized Ratio, and Glasgow Coma Scale
CI: confidence interval
CPP: cerebral perfusion pressure
CT: computed tomography
GCS: Glasgow Coma Scale
ICP: intracranial pressure
IMPACT: International Mission on Prognosis and Analysis of Clinical Trials in TBI
INR: international normalized ratio
ISS: Injury Severity Score
IV: intravenously
LHSC: London Health Sciences Centre
MAIS: Maximum Abbreviated Injury Scale
NaCl: sodium chloride
NLR: neutrophil-to-lymphocyte ratio
OR: odds ratio
PPT: partial thromboplastin time
ROC: receiver operating characteristic
SE: standard error
sTBI: severe traumatic brain injury
TBI: traumatic brain injury
